# Normal ambulatory blood pressure in young adults with 21-hydroxylase enzyme deficiency undergoing glucocorticoid replacement therapy

**DOI:** 10.20945/2359-3997000000504

**Published:** 2022-08-04

**Authors:** Juliano Henrique Borges, Daniela Albiero Camargo, Leticia Esposito Sewaybricker, Renata Isa Santoro, Daniel Minutti de Oliveira, Sofia Helena Valente de Lemos-Marini, Bruno Geloneze, Gil Guerra-Júnior, Ezequiel Moreira Gonçalves

**Affiliations:** 1 Universidade Estadual de Campinas Faculdade de Ciências Médicas Centro de Investigação em Pediatria Campinas SP Brasil Laboratório de Crescimento e Desenvolvimento (LabCreD), Centro de Investigação em Pediatria (CIPED), Faculdade de Ciências Médicas (FCM), Universidade Estadual de Campinas (Unicamp), Campinas, SP, Brasil; 2 Unicamp FCM Departamento de Pediatria Campinas SP Brasil Departamento de Pediatria, FCM, Unicamp, Campinas, SP, Brasil; 3 Unicamp FCM Laboratório de Investigação em Metabolismo e Diabetes (LIMED) Campinas SP Brasil Laboratório de Investigação em Metabolismo e Diabetes (LIMED), FCM, Unicamp, Campinas, SP, Brasil

**Keywords:** Cardiovascular risk, congenital adrenal hyperplasia, hydrocortisone, hypertension

## Abstract

**Objective:**

Herein, we compared ambulatory blood pressure (ABP) between young adults with congenital adrenal hyperplasia (CAH) due to 21-hydroxylase enzyme (21OHase) deficiency and a control group. Additionally, we analyzed correlations between the glucocorticoid dose and androgen levels and ABP parameters.

**Subjects and methods:**

This case-control study included 18 patients (6 males and 12 females) and 19 controls (8 males and 11 females) matched by age (18-31 years). ABP monitoring was used to estimate blood pressure (BP) over a 24-h period.

**Results:**

No difference was noted between patients and controls in terms of systolic BP (males, 115.5 ± 5.6 vs. 117.0 ± 9.3, P = 0.733; and females, 106.4 ± 7.9 vs. 108.4 ± 7.6, P = 0.556, respectively) and diastolic BP during 24 h (males, 62.8 ± 7.5 vs. 66.2 ± 5.6, P = 0.349; and females, 62.7 ± 4.9 vs. 62.3 ± 4.9, P = 0.818, respectively). Systolic and diastolic BP and pulse pressure during daytime and nocturnal periods were similar between patients and controls. Furthermore, no differences were detected in the percentage of load and impaired nocturnal dipping of systolic and diastolic BP between patients and controls during the 24-h period. Additionally, the glucocorticoid dose (varying between r = −0.24 to 0.13, P > 0.05) and androgens levels (varying between r = 0.01 to 0.14, P > 0.05) were not associated with ABP parameters.

**Conclusion:**

No signs of an elevated risk for hypertension were observed based on ABP monitoring in young adults with CAH attributed to 21OHase deficiency undergoing glucocorticoid replacement therapy.

## INTRODUCTION

Excessive glucocorticoid levels, as observed in Cushing's syndrome (endogenous) or due to pharmacological administration (exogenous), have been associated with hypertension in at least 70% and 20% of patients, respectively ( 1 ). Glucocorticoid and mineralocorticoid replacement therapy, when required, needs to be maintained throughout life and is designed to prevent adrenal crisis and suppress excess androgen production in patients with congenital adrenal hyperplasia (CAH) attributed to 21-hydroxylase enzyme (21OHase) deficiency ( 2 ).

According to previous reviews ( 3 , 4 ), an elevated risk of hypertension is evident in patients with CAH at different ages. However, little is known regarding the long-term impact of glucocorticoids, as well as that of regulated androgen levels, on ambulatory blood pressure (ABP) monitored during a 24-h period in young adults with CAH due to 21OHase deficiency. Elevated 24-h blood pressure (BP) has been associated with cardiovascular disease (CVD) events and mortality and exhibits a stronger association with CVD than clinical BP levels ( 5 ). It is crucial to investigate any potential detrimental effects on BP in young adults with CAH to ensure quality of life and longevity. Therefore, knowledge on how to avoid or reduce hypertension status in individuals with CAH is necessary.

In the present study, we compared ABP levels between young adults with CAH due to 21OHase deficiency and a control group. In addition, we analyzed correlations between the glucocorticoid dose and androgen levels and ABP parameters.

## SUBJECTS AND METHODS

### Participants and study design

Patient recruitment has been previously described ( 6 ). This observational and case-control study enrolled 18 young adults with CAH due to 21OHase deficiency, who were Caucasians and non-smokers; among these, 6 were males (age range: 17.8 to 27.7 years) and 12 were females (age range: 18.2 to 29.9 years). The patients exhibited a stable weight and took no medication known to affect glucose metabolism other than glucocorticoids for the last 3 months. The diagnosis of 21OHase deficiency-induced CAH was confirmed by clinical assessment, hormonal levels, and molecular analysis of the *CYP21A2* gene (OMIM *613815) ( Supplementary Table 1 ) ( 7 – 10 ). Seven patients with CAH presented with a salt-wasting phenotype and 11 with a simple virilizing phenotype.

The control group was recruited from the State University of Campinas (Unicamp), Campinas, Brazil, and comprised healthy subjects. In total, 8 males (age range: 20.9 to 28.0 years) and 11 females (age range: 22.9 to 31.1 years) were recruited and matched with the CAH group for age range, sex, ethnicity, and physical activity levels. Exclusion criteria for the control group included impaired fasting glucose, glucose intolerance, type 2 diabetes mellitus, thyroid dysfunction, severe systemic disease, and severe mental or psychiatric disturbance. In addition, non-Caucasians and smokers were excluded.

All recruited participants underwent basal clinical and laboratory assessments and body composition analysis over two days. Assessments were performed at the Metabolic Clinical Research Unit (at the Laboratory of Investigation of Metabolism and Diabetes) and in the Laboratory of Growth and Development at the School of Medical Sciences of Unicamp, Campinas, SP, Brazil. The procedures were approved by the Ethics Committee of Clinical Hospital at Unicamp, Campinas, Brazil (Certificate of Presentation for Ethical Appreciation, CAAE number 0558.0.146.000-07) (number 768/2007) in accordance with the Declaration of Helsinki for studies involving humans. Informed consent was obtained from all the subjects.

### Glucocorticoid replacement therapy

Enrolled patients were treated on an outpatient basis for approximately 22 years by the same group of physicians. Glucocorticoids were used according to doses prescribed in the last year ( Table 1 ) and were recorded as hydrocortisone equivalents (HC-equivalent), i.e., 20 mg hydrocortisone = 5 mg prednisone = 0.75 mg dexamethasone = 2 mg fludrocortisone, to standardize intake doses ( 11 ). The daily HC-equivalent dose was indexed using the body surface area (m^2^), according to the equation of Du Bois and Du Bois ( 12 ).

**Table 1:** Detailed pharmacologic therapy of patients with CAH

Patient	Sex	BMI (kg/m^2^)	Phenotype	Daily glucocorticoid therapy	Daily mineralocorticoid therapy	HC-equivalent dose (mg/m^2^/day)
1	M	27.3	SV	HC + DXM	0.050 mg	9.9
2	M	26.6	SW	HC + DXM	0.100 mg	13.6
3	M	25.9	SV	PRD		15.7
4	M	24.0	SV	HC + DXM		9.7
5	M	25.1	SW	HC + DXM	0.050 mg	13.2
6	M	29.3	SV	PRD		16.7
7	F	16.8	SV	HC + DXM	0.050 mg	12.4
8	F	19.5	SW	HC	0.050 mg	17.0
9	F	33.2	SV	HC + DXM		7.6
10	F	21.9	SW	HC + DXM	0.050 mg	12.5
11	F	24.1	SW	HC	0.050 mg	17.3
12	F	22.3	SW	HC	0.100 mg	23.6
13	F	26.9	SV	HC + DXM		11.1
14	F	19.2	SV	HC + DXM		13.7
15	F	29.3	SV	PRD		19.8
16	F	25.0	SW	HC + DXM		8.6
17	F	34.0	SV	HC + DXM	0.025 mg	8.9
18	F	26.7	SV	PRD		19.3

CAH: congenital adrenal hyperplasia; BMI: body mass index; M: male; F: female; SW: salt-wasting; SV: simple virilizing; HC: hydrocortisone; DXM: dexamethasone; PRD: prednisone.

### Physical activity level

The physical activity level (PAL) was estimated using the International Physical Activity Questionnaire-Short Form. The intensity levels of physical activity (vigorous or moderate intensity) and walking and sitting times were recorded over the last seven days. The metabolic equivalent of minutes per week was used ( 13 ). Participants were categorized as active or inactive ( 6 ).

### Percentage of fat analysis

Whole-body weight and fat mass were estimated using dual-energy X-ray absorptiometry (iDXA with software enCORE™ 2011 version 13.60, GE Healthcare Lunar, Madison, WI, USA). In addition, the percentage fat mass (% fat) was analyzed. The coefficient of variation for fat mass was 0.9% ± 0.9% at our laboratory.

### Biochemical analyses

Blood samples were collected after overnight fasting for biochemical assessments. Blood samples were also collected from patients before the first administration of glucocorticoids in the morning. Plasma glucose levels were measured using an enzymatic hexokinase method. The ion-selective electrode method was used to analyze sodium and potassium concentrations. In addition, serum insulin, high-sensitivity C-reactive protein (hsCRP), adrenocorticotropic hormone (ACTH), androstenedione, total testosterone, 17-hydroxyprogesterone (17OHP), and renin levels were measured using the Modular E170 automated chemiluminescent immunometric method (Roche Diagnostics, Indianapolis, IN, USA) with an intra-assay coefficient of variation of <3.5%. Insulin and glucose values were used to calculate the homeostasis model assessment index for insulin resistance (HOMA-IR) ( 14 ).

### Ambulatory blood pressure monitoring

Subjects underwent a 24-h ABP recording using the Spacelabs 90207 ambulatory monitor (Spacelabs Medical, Issaquah, WA, USA). The BP system was programmed to measure every 20 min, from 06:00 to 22:00 h, and every 1 h, from 22:00 to 06:00. Self-reported sleep-wake times were used to divide 24-h ABP monitoring data into daytime and nocturnal periods. Pulse pressure (PP) was calculated based on the difference between systolic and diastolic BP ( 15 ). The percentage of BP load (readings above the limit values) was provided by the manufacturer of the ambulatory monitor. Percent dipping was calculated for both average systolic and diastolic BP using the following formula: [(daytime BP - nocturnal BP)/daytime BP] × 100. Each subject was categorized as a “dipper” (decrease in average systolic and diastolic BP ≥ 10% during sleep) or a “non-dipper” (decrease < 10%) ( 16 ). A hypertension state for 24-h ABP monitoring was defined according to the Brazilian Guidelines of Hypertension – 2020 ( 17 ): 24-h systolic BP ≥ 130 mmHg and/or 24-h diastolic BP ≥ 80 mmHg; daytime systolic BP ≥ 135 mmHg and/or daytime diastolic BP ≥ 85 mmHg; nocturnal systolic BP ≥ 120 mmHg and/or nocturnal diastolic BP ≥ 70 mmHg.

### Statistical analyses

Descriptive data analysis values are presented as mean and standard deviation, while the Shapiro-Wilk test was used to verify data normality. Unpaired Student's t-test or Mann-Whitney U-test was used to compare differences between CAH and control groups for clinical markers, hormonal levels, and ABP parameters. Additionally, the Chi-squared test was used to verify the homogeneous distribution of inactive and active lifestyles between PAL groups, as well as the distribution of dipper and non-dipper individuals between groups in terms of systolic and diastolic BP nocturnal dipper. Finally, multiple linear correlation was used to identify correlations between the glucocorticoid dose and androgens levels and ABP parameters after adjusting by sex. Data analyses were performed using SPSS version 16.0 (Statistical Package for the Social Sciences, Chicago, IL, USA). The significance level was set at α ≤ 0.05.

## RESULTS

Table 2 presents the characteristics of patients and controls. Patients showed an average duration of glucocorticoid therapy of 22.0 ± 3.5 years, at an average of 13.9 ± 4.4 mg/m^2^/day of HC-equivalent dose. No differences were observed between patients and controls for weight, glucose, insulin, HOMA-IR, hsCRP, potassium, sodium, ACTH, androstenedione, and total testosterone concentrations. Patients of both sexes exhibited a significantly shorter height than control subjects ( *P* < 0.05). Male patients, but not females, demonstrated higher body mass index and % fat than controls. However, female patients, but not males, were younger and presented elevated 17OHP and renin levels when compared with control subjects ( *P* < 0.05). Despite the lack of differences in the distribution of PAL between the two groups, an active lifestyle was observed in approximately 83% and 42% of patients and 87% and 73% of controls (males and females, respectively).

**Table 2:** Characteristics of patients with CAH and control subjects (Mean ± SD)

	Males					Females				
	n	CAH	n	Control	*P* -value	n	CAH	n	Control	*P* -value
Age (years)	06	24.0 ± 3.6	08	24.3 ± 2.1	0.854	12	23.5 ± 3.3	11	26.5 ± 2.7	**0.027**
Weight (kg)	06	70.4 ± 6.3	08	70.6 ± 9.1	0.968	12	60.3 ± 13.5	11	59.4 ± 10.6	0.928
Height (cm)	06	163.3 ± 7.1	08	173.9 ± 8.2	**0.027**	12	155.7 ± 6.7	11	162.2 ± 7.6	**0.040**
BMI (kg/m^2^)	06	26.4 ± 1.8	08	23.3 ± 1.9	**0.010**	12	24.9 ± 5.4	11	22.5 ± 2.9	0.197
% fat	06	28.3 ± 8.3	08	19.2 ± 4.7	**0.023**	10	36.8 ± 7.0	11	31.7 ± 5.8	0.080
Duration of treatment (years)	06	19.7 ± 3.3	-	-	-	12	23.1 ± 3.2	-	-	-
HC-equivalent dose (mg/m^2^/day)	06	13.1 ± 2.9	-	-	-	12	14.3 ± 5.1	-	-	-
Glucose (mg/dL)	06	86.7 ± 9.6	07	84.0 ± 3.7	0.545	12	84.1 ± 9.6	11	83.6 ± 5.8	0.895
Insulin (uUI/mL)	06	5.6 ± 2.5	07	5.9 ± 3.6	0.871	10	12.7 ± 13.2	11	7.4 ± 4.1	0.173
HOMA-IR	06	1.19 ± 0.49	07	1.22 ± 0.74	0.942	10	2.80 ± 3.20	11	1.55 ± 0.94	0.282
hsCRP (mg/dL)	06	0.18 ± 0.23	07	0.17 ± 0.23	0.295	12	0.19 ± 0.19	11	0.44 ± 0.87	0.379
Potassium (mEq/L)	06	4.0 ± 0.3	07	4.1 ± 0.1	0.417	11	4.1 ± 0.3	11	4.1 ± 0.3	0.726
Sodium (mEq/L)	06	141.5 ± 1.4	07	141.1 ± 1.3	0.646	11	140.4 ± 2.9	11	139.4 ± 2.1	0.332
ACTH (pg/mL)	06	111.8 ± 204.1	07	35.0 ± 18.0	1.000	12	32.7 ± 45.0	10	28.1 ± 13.3	0.254
Androstenedione (ng/mL)	06	2.5 ± 3.2	07	1.9 ± 0.6	0.628	12	2.3 ± 1.9	11	2.0 ± 1.0	1.000
Total testosterone (ng/mL)	06	5.19 ± 1.75	07	6.36 ± 1.09	0.167	12	0.29 ± 0.30	11	0.25 ± 0.16	0.525
17OHP (ng/mL)	06	24.0 ± 36.3	07	2.0 ± 0.5	0.366	12	15.1 ± 22.4	09	0.6 ± 0.2	0.015
Renin (pg/mL)	06	30.8 ± 12.8	05	34.9 ± 21.7	0.706	11	28.5 ± 24.2	05	7.0 ± 1.9	0.013
PAL (Inactive/Active lifestyle)	06	01/05	08	01/07	1.000	12	07/05	11	03/08	0.214

% fat: percentage fat mass; 17OHP: 17-hydroxyprogesterone; ACTH: adrenocorticotropic hormone; BMI: body mass index; CAH: congenital adrenal hyperplasia; HC: hydrocortisone; HOMA-IR: homeostasis model assessment for insulin resistance index; hsCRP: high-sensitivity C-reactive protein; PAL: physical activity level.

Values in boldface indicate significant differences ( *P* < 0.05).

Table 3 presents the ABP values of patients and controls. The mean duration of ABP monitoring was 23.5 ± 1.0 h, reaching satisfactory readings mean of 75.4 ± 19.4%. No differences were observed in terms of systolic and diastolic BP and PP between patients and controls of both sexes during the 24-h, daytime, and nocturnal periods. In addition, no difference was detected in the percentage load and nocturnal dipping of systolic and diastolic BP between patients and controls of both sexes. All patients and controls demonstrated a normal BP status. However, only 25% and 12.5% of male controls presented altered systolic BP during the 24-h and nocturnal periods, respectively. In addition, only 9.1% of female controls exhibited altered systolic BP during the nocturnal period.

**Table 3:** Ambulatory blood pressure values of patients with CAH and control subjects (Mean ± SD)

		Males					Females				
		n	CAH	n	Control	*P* -value	n	CAH	n	Control	*P* -value
Mean											
	24-h SBP (mmHg)	06	115.5 ± 5.6	08	117.0 ± 9.3	0.733	12	106.4 ± 7.9	11	108.4 ± 7.6	0.556
	24-h DBP (mmHg)	06	62.8 ± 7.5	08	66.2 ± 5.6	0.349	12	62.7 ± 4.9	11	62.3 ± 4.9	0.818
	Daytime SBP (mmHg)	06	117.5 ± 5.3	08	118.1 ± 9.7	0.889	12	108.5 ± 8.7	11	109.4 ± 7.6	0.802
	Daytime DBP (mmHg)	06	64.5 ± 8.1	08	67.1 ± 6.4	0.511	12	65.1 ± 5.2	11	63.7 ± 4.7	0.518
	Nocturnal SBP (mmHg)	06	107.7 ± 9.5	08	108.5 ± 8.8	0.869	12	100.1 ± 8.5	11	104.6 ± 9.7	0.245
	Nocturnal DBP (mmHg)	06	55.7 ± 6.6	08	59.0 ± 4.0	0.263	12	55.5 ± 5.9	11	55.8 ± 6.0	1.000
Pulse pressure											
	24-h PP (mmHg)	06	53.0 ± 4.4	08	51.0 ± 5.8	0.495	12	43.7 ± 4.8	11	45.9 ± 5.2	0.309
	Daytime PP (mmHg)	06	53.0 ± 4.4	08	51.2 ± 5.9	0.555	12	43.6 ± 4.7	11	45.4 ± 5.0	0.362
	Nocturnal PP (mmHg)	06	51.8 ± 5.4	08	49.6 ± 6.2	0.498	12	44.6 ± 5.4	11	48.9 ± 6.2	0.088
Load											
	SBP (%)	06	5.7 ± 9.2	08	13.8 ± 15.1	0.228	12	1.7 ± 3.0	11	3.9 ± 4.6	0.190
	DBP (%)	06	8.3 ± 8.2	08	7.0 ± 6.7	1.000	12	2.2 ± 2.5	11	2.2 ± 2.7	0.740
Nocturnal dipping											
	SBP (%)	06	8.5 ± 5.0	08	7.8 ± 8.9	0.867	12	7.7 ± 6.0	11	4.4 ± 5.0	0.180
	DBP (%)	06	13.7 ± 2.5	08	11.6 ± 9.0	0.599	12	14.6 ± 8.3	11	12.5 ± 6.3	0.505

SBP: systolic blood pressure; DBP: diastolic blood pressure; PP: pulse pressure.

**Figure 1:**
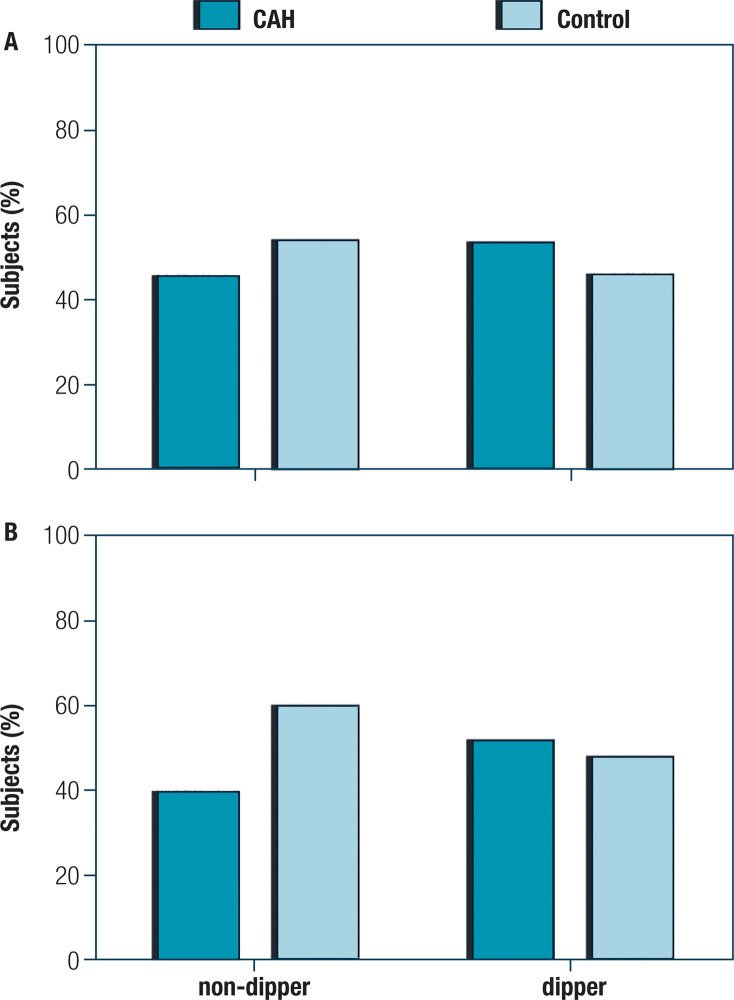
Percentage of non-dipper and dipper cases in patients with congenital adrenal hyperplasia (CAH) and control subjects. A) systolic blood pressure; and B) diastolic blood pressure.

The percentages of non-dipper and dipper cases among patients and controls are shown in Figure 1 . On analyzing nocturnal dipping of systolic BP, 45.8% of non-dipper individuals were patients and 54.2% were controls, whereas 53.8% of dipper individuals were patients and 46.2% were controls ( *P* = 0.737) ( Figure 1A ). Similar to the nocturnal dipping of diastolic BP, Figure 1B shows that 40.0% of non-dipper individuals were patients and 60.0% were control subjects, whereas 51.9% of dipper individuals were patients and 48.1% were controls ( *P* = 0.714).

Table 4 presents the adjusted sex correlation between ABP values and the glucocorticoid dose and androgen levels. The analysis demonstrated that systolic and diastolic BP during a 24-h, daytime, and nocturnal period did not correlate with the HC-equivalent dose (varying between *r* = −0.24 to 0.13, *P* > 0.05) and androstenedione levels (varying between *r* = 0.01 to 0.14, *P* > 0.05).

**Table 4:** Correlation between ambulatory blood pressure values and glucocorticoid dose and androgen levels of patients with CAH – adjusted by sex ( *n* = 18)

	HC-equivalent dose		Log Androstenedione	
	Partial *r*	*P* -value	Partial *r*	*P* -value
24-h SBP	-0.09	0.731	0.14	0.433
24-h DBP	0.04	0.882	0.02	0.900
Daytime SBP	-0.02	0.924	0.12	0.480
Daytime DBP	0.13	0.616	0.01	0.976
Nocturnal SBP	-0.24	0.349	0.12	0.496
Nocturnal DBP	-0.15	0.558	0.02	0.892

SBP: systolic blood pressure; DBP: diastolic blood pressure; HC: hydrocortisone.

## DISCUSSION

The main results of the present study were normal values for parameters of 24-h ABP monitoring in patients with 21OHase deficiency-induced CAH. Additionally, the dose of glucocorticoid therapy and androgen levels were not associated with ABP parameters.

ABP is highly recommended during the 24-h period and is an assessment that may precede the development of elevated clinical BP levels ( 18 ). Few studies have investigated ABP during a 24-h period in young adults with CAH when compared with healthy individuals ( 19 , 20 ). Previously published reviews have suggested that patients with CAH at different ages exhibit elevated BP ( 3 , 4 ). However, consistent with the findings of Falhammar and cols. ( 20 ), but not Mooij and cols. ( 19 ), we detected normal values for ABP during a 24-h period in young adults with CAH. In addition, we noted normal ABP values during daytime and nocturnal periods. Although some male patients presented with elevated % fat, and female patients presented with altered renin levels, these changes did not influence BP. Thus, it can be suggested that patients in this study presented with no elevated risk of hypertension.

Patients showed ambulatory PP within the normal range during 24-h, daytime, and nocturnal periods. PP reflects the stiffening of large arteries and can be associated with several CVD risk factors ( 15 ). In addition, the percentage of systolic and diastolic BP loads and nocturnal dipping exhibited normal values during the 24-h period. ABP load is defined as the proportion of elevated systolic and diastolic BP over a 24-h period, representing chronic pressure overload that induces myocardial and vascular damage associated with the hypertensive disease process ( 21 ). Furthermore, accumulating evidence suggests that nocturnal ABP is a better predictor of outcome than daytime ABP in patients with hypertension ( 22 ). Additionally, nocturnal dipping of systolic and diastolic BP by more than 10% of daytime values can exert beneficial effects by delaying or preventing the development of cardiac left ventricular hypertrophy ( 16 ). Thus, persistently elevated circulating glucocorticoids have been shown to induce a non-dipping BP profile ( 23 ). We also observed no difference in the distribution of non-dipper and dipper individuals between patients and controls. To the best of our knowledge, this is the first analysis of PP, load, and nocturnal dipping in young adults with CAH undergoing glucocorticoid replacement therapy when compared with healthy individuals. Accordingly, it can be postulated that the arterial elastic properties and structures were preserved in patients investigated in the present study.

The mechanism underlying glucocorticoid-induced hypertension remains unknown ( 24 , 25 ). Saruta ( 25 ) has suggested that multiple factors are involved in glucocorticoid-induced hypertension in humans and animals: activation of the renin-angiotensin system due to an increase in plasma renin substrate, reduced activity of depressor systems, including the kallikrein-kinin system, prostaglandins, and the endothelium-derived relaxing factor nitric oxide, and increased pressor responses to angiotensin II and norepinephrine. Völkl and cols. ( 26 ) have reported a positive correlation between the HC-equivalent dose and some BP parameters. However, we observed that the glucocorticoid dose or androgen levels were not associated with systolic and diastolic BP during the 24-h, daytime, and nocturnal periods. In the present study, patients presented a well-controlled state, with no signs of androgen or cortisol excess, and a healthy metabolic state. Metwalley and cols. ( 27 ) have revealed that poorly controlled patients with CAH exhibit elevated systolic and diastolic BP when compared with well-controlled patients. Furthermore, the average HC-equivalent dose in the present study was 13.9 ± 4.4 mg/m^2^/day, approaching the normal physiological cortisol production rate (estimated to be 9-11 mg/m^2^/day) ( 28 ). Thus, we believe that the outcomes are important findings, demonstrating the optimization of the clinical treatment in patients with CAH.

However, it is important to list some limitations of the present study, such as the small sample size, different phenotypes, and types/doses of glucocorticoid intake. Furthermore, glucocorticoids and mineralocorticoids were calculated according to the doses prescribed in the previous year and may not represent lifetime exposure. However, a significant strength of our study is that enrolled patients were undergoing clinical treatment for approximately 22 years on an outpatient basis by a single group of physicians dedicated to their specialized care. Additionally, patients in this study presented an optimum adherence to medical appointments and pharmacological treatment. Thus, these factors may explain the optimal hormonal control with no signs of androgen or cortisol excess. Consequently, the well-controlled CAH may explain the outcomes of the present study, which is in contrast with that reported in the literature ( 3 , 4 , 27 ). Despite some limitations, determining behavioral patterns for patients with CAH is a multifactorial challenge, given the variability of CAH abnormalities, dose, type, and duration of pharmacologic therapy, age range, and physical activity. These findings could help endocrinologists and cardiologists select an adequate pharmacologic therapy for young adults, thereby improving the patients’ quality of life.

In conclusion, we observed no signs of elevated risk for hypertension after ABP monitoring in young adults with CAH attributed to 21OHase deficiency. Accordingly, adequate pharmacologic therapy and follow-up may help avoid impaired BP, thus decreasing the risk of CVD and increasing the longevity of patients with CAH.

## Appendix

**Supplementary Table 1:** Molecular analysis of patients to confirm and determine phenotype form of congenital adrenal hyperplasia ( *n* = 18)

Patient	Father (1)	Mutation gene 1	Mother (2)	Mutation gene 2	Predicted gene
1	c.515T>A / p.Ile172Asn	SV	c.320T>G / p.Leu107Arg	SW	SV
2	c.[920_921insT;952C>T;1066C>T] / p.[Leu308Phefs*6;Gln318*;Arg356Trp]	SW	c.[920_921insT;952C>T;1066C>T] / p.[Leu308Phefs*6;Gln318*;Arg356Trp]	SW	SW
3	c.290-2A>G / p.Tyr97Serfs*11	SW	c.515T>A / p.Ile172Asn	SV	SV
4	conversion 1	SV	c.515T>A / p.Ile172Asn	SV	SV
5	c.290-13A/C>G / p.Tyr97Serfs*11	SW	c.290-13A/C>G / p.Tyr97Serfs*11	SW	SW
6	conversion 1	SW	conversion 1	SV	SV
7	c.515T>A / p.Ile172Asn	SV	deletion 1	SW	SV
8	c.290-13A/C>G / p.Tyr97Serfs*11	SW	conversion 4	SW	SW
9	p.Gly56Arg	SV	c.290-13A/C>G / p.Tyr97Serfs*11	SW	SV
10	deletion 1	SW	deletion 1	SW	SW
11	c.1222C>T / p.Arg408Cys	SW	c.290-13A/C>G / p.Tyr97Serfs*11	SW	SW
12	c.1222C>T / p.Arg408Cys	SW	c.290-13A/C>G / p.Tyr97Serfs*11	SW	SW
13	conversion 1	SV	c.515T>A / p.Ile172Asn	SV	SV
14	c.[515T>A;841G>T] / p.[Ile172Asn;Val281Leu]	SV	conversion 1	SV	SV
15	c.290-13A/C>G / p.Tyr97Serfs*11	SW	c.515T>A / p.Ile172Asn	SV	SV
16	c.515T>A / p.Ile172Asn	SV	c.425T>C / p.Leu142Pro	SW	SV
17	c.515T>A / p.Ile172Asn	SV	c.320T>G / p.Leu108Arg	SW	SV
18	conversion 1	SV	conversion 1	SV	SV

SV: simple virilizing; SW: salt-wasting.

Conversion 1 - means that it is an allele from molecular rearrangement where a hybrid gene can be formed without loss of DNA. In the case of this conversion that I called conversion 1, the hybrid gene is formed by sequences from the pseudogene (CYP21A1P) from the 5′ promoter region to exon 1 that includes the pathogenic variation c.89C>T / p.Pro30Leu; from exon 2 to the 3′ terminal is formed by sequences of the active gene (CYP21A2). This type of rearrangement is associated with the classic non salt-wasting form of 21-hydroxylase deficiency.

Conversion 2 - means that it is an allele from molecular rearrangement where a hybrid gene can be formed without loss of DNA. In the case of this conversion that I called conversion 2, the hybrid gene is formed by sequences of the pseudogene (CYP21A1P) from the promoter region 5′ to exon 3 that includes the pathogenic variations c.290-13A/C>G / p.Tyr97Serfs*11 and c.329_336 delGAGACTAC / p.Gly110Valfs*20; from exon 4 to the 3′ terminal is formed by sequences of the active gene (CYP21A2). This type of rearrangement is associated with the classic salt-wasting form of 21-hydroxylase deficiency.

Conversion 3 - means that it is an allele from molecular rearrangement where a hybrid gene can be formed without loss of DNA. In the case of this conversion that I called conversion 2, the hybrid gene is formed by sequences from the pseudogene (CYP21A1P) from the 5′ promoter region to exon 3 that includes the pathogenic variations c.89C>T / p.Pro30Leu, c.290 -13A/C>G / p.Tyr97Serfs * 11 and c.329_336 delGAGACTAC / p.Gly110Valfs*20; from exon 4 to the 3′ terminal is formed by sequences of the active gene (CYP21A2). This type of rearrangement is associated with the classic salt-wasting form of 21-hydroxylase deficiency.

Conversion 4 - means that it is an allele from molecular rearrangement where a hybrid gene can be formed without loss of DNA. In the case of this conversion that I called conversion 2, the hybrid gene is formed by sequences of the pseudogene (CYP21A1P) from the promoter region 5′ to exon 7 that includes the pathogenic variations c.89C>T / p.Pro30Leu, c.290 -13A/C>G / p.Tyr97Serfs*11, c.329_336 delGAGACTAC / p.Gly110Valfs*20, c.515T>A / p.Ile172Asn, c.[707T>A;708C>G;710T>A;716T>A] / p. [Ile236Asn;Ile236Met;Val237Glu;Met239Lys], c.841G>T / p.Val281Leu and c.920_921insT / p.Leu308Phefs*6; from exon 8 to the 3′ terminal is formed by sequences of the active gene (CYP21A2). This type of rearrangement is associated with the classic salt-wasting form of 21-hydroxylase deficiency.

Deletion 1 - means that it is an allele from molecular rearrangement where a hybrid gene can be formed with loss of 30 kb of DNA. In the case of this deletion that I called deletion 1, the hybrid gene is formed by sequences from the pseudogene (CYP21A1P) from the 5′ promoter region to exon 7 that includes the pathogenic variations c.89C>T / p.Pro30Leu, c.290-13A/C>G / p.Tyr97Serfs*11, c.329_336 delGAGACTAC / p.Gly110Valfs*20, c.515T>A / p.Ile172Asn, c. [707T>A;708C>G;710T>A;716T>A] / p.[Ile236Asn;Ile236Met;Val237Glu;Met239Lys], c.841G>T / p.Val281Leu and c.920_921insT / p.Leu308Phefs*6; from exon 8 to the 3′ terminal is formed by sequences of the active gene (CYP21A2). This type of rearrangement is associated with the classic salt-wasting form of 21-hydroxylase deficiency.

Deletion 2 - means that it is an allele from molecular rearrangement where a hybrid gene can be formed with loss of 30 kb of DNA. In the case of this deletion that I called deletion 2, a hybrid gene is not formed, the gene present in the allele has only sequences from the pseudogene (CYP21A1P) from the promoter region 5′ to the 3′ terminal. This type of rearrangement is associated with the classic salt-wasting form of 21-hydroxylase deficiency.
